# IL-38: A new factor in rheumatoid arthritis

**DOI:** 10.1016/j.bbrep.2015.10.015

**Published:** 2015-10-31

**Authors:** Shin-ichi Takenaka, Shinjiro Kaieda, Tomotaka Kawayama, Masanobu Matsuoka, Yoichiro Kaku, Takashi Kinoshita, Yuki Sakazaki, Masaki Okamoto, Masaki Tominaga, Katsuya Kanesaki, Asako Chiba, Sachiko Miyake, Hiroaki Ida, Tomoaki Hoshino

**Affiliations:** a Department of Medicine, Division of Respirology, Neurology and Rheumatology, Kurume University School of Medicine, Kurume 830-0011, Japan; b Nagata Orthopedic Hospital, Omuta 836-0843, Japan; c Department of Immunology, Juntendo University School of Medicine, Tokyo 113-8421, Japan; d CIP, NCI-Frederick, NIH, Frederick, MD 21702, United States

**Keywords:** IL-38, IL-1 family, RA mouse model

## Abstract

The newly characterized cytokine IL-38 (IL-1F10) belongs to the IL-1 family of cytokines. Previous work has demonstrated that IL-38 inhibited *Candida albicans*-induced IL-17 production from peripheral blood mononuclear cells. However, it is still unclear whether IL-38 is an inflammatory or an anti-inflammatory cytokine. We generated anti-human IL-38 monoclonal antibodies in order to perform immunohistochemical staining and an enzyme-linked immunosorbent assay. While human recombinant IL-38 protein was not cleaved by recombinant caspase-1, chymase, or PR3 in vitro, overexpression of IL-38 cDNA produced a soluble form of IL-38 protein. Furthermore, immunohistochemical analysis showed that synovial tissues obtained from RA patients strongly expressed IL-38 protein. To investigate the biological role of IL-38, C57BL/6 IL-38 gene-deficient (^−^^/−^) mice were used in an autoantibody-induced rheumatoid arthritis (RA) mouse model. As compared with control mice, IL-38 ^(−/−)^ mice showed greater disease severity, accompanied by higher IL-1β and IL-6 gene expression in the joints. Therefore, IL-38 acts as an inhibitor of the pathogenesis of autoantibody-induced arthritis in mice and may have a role in the development or progression of RA in humans.

## Highlights

• The soluble form of IL-38 is detected in the sera obtained from of RA patients.
• IL-38 protein was highly expressed in the synovial lining of RA synovium.
• IL-38 expression was up-regulated during arthritis in mice at the mRNA level.
• IL-38 may attenuate joint inflammation by inhibiting the IL-1 induced inflammation.

## 1 Introduction

IL-38 (IL-1F10) was originally cloned as an IL-1 family cytokine, and named IL-1HY2 in 2001 [1]. A human IL-38 cDNA sequence identified from a human fetal skin library encodes a 152-amino-acid precursor. Human IL-38 has 4 exons, and the IL-38 gene is located in the IL-1 family cluster on chromosome 2 next to the genes encoding IL-1 receptor antagonist (IL-1Ra) and IL-36 receptor antagonist (IL-36Ra). IL-38 shares 37% homology with IL-1Ra, 43% homology with IL-36Ra and has a three-dimensional structure similar to IL-1Ra [2]. Immunohistochemical analysis using a polyclonal antibody against an IL-38-origin synthetic peptide (CTLPNRGLDRTKVP) has shown that the IL-38 protein is expressed in the basal epithelia of human skin and in proliferating B cells of the tonsil [1]. Similarly to IL-1α, IL-1β, IL-1Ra, IL-18, IL-33, IL-36, and IL-37, IL-38 lacks a signal peptide. The natural N terminus of IL-38 is still unknown, and there is no caspase-1 consensus cleavage site [2]. A recent study has demonstrated that IL-38 inhibited *Candida albicans*-induced IL-17, as well as IL-22 production from peripheral blood mononuclear cells (PBMC). In contrast, LPS-induced IL-6 production was significantly higher in the presence of IL-38 [3]. The precise biological roles of IL-38 are still unknown.

## 2 Materials and methods

### 2.1 Recombinant human IL-38 protein

The full-length human IL-38 cDNA [1] with a His tag at the C-terminal, converted to an *Escherichia coli*-type codon, was synthesized in accordance with the GenBank sequence (BC103968.1). The synthesized human IL-38 cDNA was subcloned into the pET28a (+) expression vector (Novagen, Tokyo, Japan). *E. coli* (BL21DE3) was transformed with pET28a/human IL-38. Recombinant human IL-38 was isolated as reported previously [4]. The full-length human IL-38 cDNA with a His tag at the C-terminal was subcloned into the pPSC8 expression vector (Protein Sciences Corporation, Meriden, CT, USA), and designated as pPSC8/human IL-38. We also isolated recombinant human IL-38 protein from SF9 cells co-transfected with baculovirus AcNPV and pPSC8/human IL-38.

### 2.2 Human IL-38 cDNA-transfected cells

The synthesized human IL-38 cDNA was subcloned into the pEF1α-IRES vector (Clontech Laboratories, Inc., Mountain View, CA, USA). The pEF1α-IRES/human IL-38 plasmid was transfected into 293T cells (human embryonic kidney cell line) using FuGENE® 6 DNA transfection regent (Promega KK, Tokyo, Japan). The pEF1α-IRES/human IL-38 plasmid was also transfected into the murine myeloma cell line P815 using a Gene Pulser II (Bio-Rad, Hercules, CA), as reported previously [5].

### 2.3 Establishment of an anti-human IL-38 polyclonal antibody and monoclonal antibody

Specific pathogen-free (Japanese White) rabbits were immunized with recombinant human IL-38 protein, and antisera were obtained. Purified rabbit anti-human IL-38 polyclonal antibody was generated from the antisera in our laboratory, as reported previously [5], [6]. An anti-human IL-38 monoclonal antibody (mAb) was established by fusion of the mouse myeloma cell line X-63・Ag8/653 with spleen cells isolated from a BALB/c mouse immunized with the recombinant human IL-38 protein, as reported previously [5], [6]. We generated ascites using this cell line, and purified the antibody using a protein G column. The purified mAb was labeled with FITC, as reported previously [7].

### 2.4 Establishment of a human IL-38 sandwich enzyme-linked immunosorbent assay system

A human IL-38 sandwich enzyme-linked immunosorbent assay (ELISA) system was established, as reported previously. Briefly, a mouse anti-human IL-38 primary mAb (clone H160A) dissolved at 2 μg/mL in phosphate-buffered saline (PBS) was dispensed into enzyme-linked immunosorbent assay (ELISA) plates in aliquots of 100 μL/well and left undisturbed overnight at 4 °C to allow it to become immobilized. The plates were then washed three times with 200 μL of Quantikine Wash Buffer 1 (R&D Systems, Minneapolis, MN, USA), and 200 μL/well of 10% Block Ace blocking solution (Nakarai Tesque, Kyoto, Japan) was added and left for at least one hour at room temperature to prevent nonspecific adhesion of the secondary antibody to the plates. The plates were then washed three times. Human serum samples were then aliquoted at 100 μL/well. Recombinant human IL-38 protein diluted to 600, 300, 150, 75, 37.5, 18.75 and 9.375 ng/mL was used as the standard. After 2 h of incubation at room temperature, each well was washed three times. Next, 1 μg/mL biotin-labeled mouse anti-human IL-38 secondary mAb (clone H127C) was dispensed at 100 μL/well, followed by incubation for 90 min at room temperature, and then each well was washed four times. This was followed by addition of 100 μL of 0.5 μg/mL streptavidin-bound horseradish peroxidase (Millipore, Tokyo, Japan) to each well, and the plates were left undisturbed for 30 min at room temperature. Each well was then washed three times. A color development substrate solution (ELISA POD Substrate TMB kit; Nakarai Tesque) was then added at 100 μl/well, and the plates were left undisturbed for 30 min at room temperature, followed by addition of a stop solution (1 M sulfuric acid; Wako, Tokyo, Japan) at 100 μL/well to stop the enzyme reaction. The amounts of the human IL-38 protein were determined by measuring the absorbance at 450 nm. The limit of sensitivity of this ELISA system was <9.35 ng/mL.

### 2.5 Protease reaction and Western blotting analysis

Digestion of recombinant human IL-38 and pro-IL-1β protein (Sino Biological Inc., Beijing, China) by recombinant caspase 1, chymase, and PR3 (Sigma, St. Louis, MO, USA) was performed as reported previously [8], [9]. Then, we performed Western blotting analysis to evaluate the reactions by using a rabbit anti-human IL-38 polyclonal antibody (established in our laboratory) and rabbit anti-human pro-IL-1β (Santa Cruz Biotechnology, Dallas, TX, USA).

### 2.6 Human subjects

Synovial tissue was obtained from knee joints of patients with rheumatoid arthritis (RA) (*n*=7) and osteoarthritis (OA) (*n*=2), who underwent knee surgery at the Nagata Orthopedic Hospital (Omuta, Japan). All samples were fixed in formalin and embedded in paraffin. Serum samples were obtained from 137 RA patients (22 males and 115 females) and 26 OA patients (5 males and 21 females), who had been consecutively monitored at the Kurume University Hospital or the Kurume University Medical Center (Kurume, Japan) from 2005 to 2010. Serum samples were also obtained from 56 age-matched healthy volunteers (33 males and 23 females). Sample collection and all procedures were approved by the ethics committee of Kurume University in accordance with the ethical standards of the Helsinki Declaration of 1975. Informed consent was obtained from all patients and healthy volunteers.

### 2.7 Histological examinations and immunohistochemical staining

Immunohistochemical staining was performed as reported previously [5], [6], [10]. Briefly, synovial tissues were fixed with 10% formalin and embedded in paraffin wax. Serial sections (4 μm thick) were cut from paraffin-embedded tissues, placed on poly-l-lysine-coated slides, and then incubated overnight at 55–60 °C. Deparaffinized sections were autoclaved for 3–4 min in 10 mM citric acid buffer (pH 6.0). Deparaffinized sections were immunostained with anti-human IL-38 mAb (clone H127C [mouse IgG2b], 0.5 μg/mL) for 60–90 min at room temperature. Positive reactivity was identified using biotin-labeled goat anti-mouse and rabbit IgG, peroxidase-streptavidin and 3-3′-diaminobenzidine-4HCl (Dako, Kyoto, Japan). Anti-human IL-38 clone H127C was also used for intracellular staining for flow cytometry (data not shown).

### 2.8 IL-38-deficient mice

IL-38-deficient mice (Il1f10tm1Lex/Mmucd), backcrossed 9 times with C57BL/6NCrl (B6) mice, were obtained from the Mutant Mouse Regional Resource Center, University of California Davis (West Sacramento, CA). Age-matched female B6 wild-type (WT) mice, purchased from Charles River Japan (Yokohama, Japan), were used as controls. All procedures were approved by the Committee on the Ethics of Animal Experiments, Kurume University (Approval no. 2013-243). Animal care was provided in accordance with the procedures outlined in the “Principles of laboratory animal care” (National Institutes of Health Publication no.86-23, revised 1985). All efforts were made to minimize the suffering of animals used in this study.

### 2.9 Experimental RA mouse model

Pro-arthritic serum was isolated from K/BxN mice as described previously [11], [12]. K/BxN arthritis was induced by intraperitoneal injection of 150 μL K/BxN serum on day 0 of each experiment. Arthritis was graded using a 0–16 clinical scale (0–4) per paw, as described previously [13]. Histological assessment was performed on paraffin-embedded 4-μm sections stained with hematoxylin and eosin, and synovial inflammation and bone erosion were graded in a blinded manner emplying a 0–5 scale, in accordance with an established system [14].

### 2.10 Real-time quantitative RT-PCR

Total RNA was isolated from mouse ankle joints using the RNeasy minikit (Qiagen, Tokyo, Japan). Total RNA was extracted from mouse ankle joints as described previously [15], [16]. Briefly, the skin around the ankle joints was removed, and tissue from the distal tibia to the mid paw was carefully collected to avoid contamination with bone marrow. The harvested ankles were immersed in RNAlater (Qiagen) to minimize degradation of RNA. The samples were treated with proteinase K (55 °C, 30 min), and cells were disrupted with Buffer RLT lysis buffer (Qiagen). For the follow-up real-time-quantitative PCR (qPCR) assays, purified RNA was converted in each instance into cDNA using a Quantitect reverse transcription kit (Qiagen). qPCRs were performed with SYBR Green Mastermix (Qiagen) using primers for mouse and human β-actin and IL-38 and mouse IL-1β and IL-6 (Qiagen) on an Mx3000p PCR machine (Stratagene, La Jolla, CA). Relative expression was calculated using the comparative threshold cycle method, as reported previously [17].

### 2.11 Statistical analysis

Results were expressed as means±standard error of the mean (SEM). *P* values were calculated by Mann–Whitney U-test. Differences were considered significant at *P*<0.05.

## 3 Results

### 3.1 Characteristics of human IL-38

**Fig. 1:**
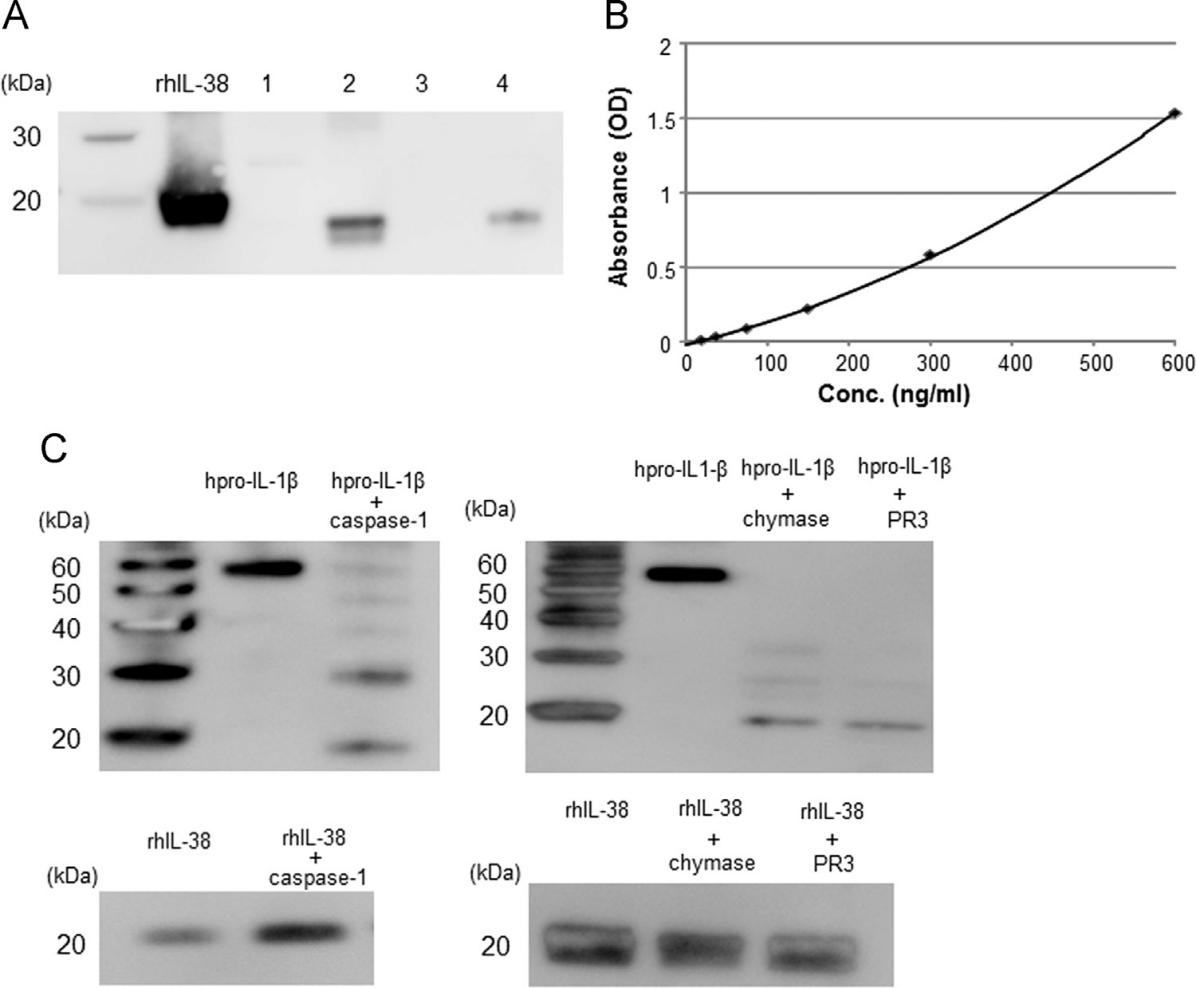
Characteristics of human IL-38. (A) Western blotting analysis using anti-human IL-38 mAb (H127C) showed that human IL-38 cDNA-transfected 293T cells expressed approximately 18 kDa IL-38 protein in the cells and supernatants. Line 1: Cell lysate of vector-transfected 293T cells. Line 2: Cell lysate of human IL-38 cDNA-transfected-transfected 293T cells. Line 3: Supernatant of vector-transfected 293T cells. Line 4: Supernatant of human IL-38 cDNA-transfected-transfected 293T cells. As control, recombinant human IL-38 (rhIL-38) protein with His tag at C-terminal was used. (B) Standard curve of human IL-38 sandwich ELISA using anti-human IL-38 mAbs (H127C and H160A). (C) Caspase-1, chymase, and PR3 can cleave recombinant human pro-IL-1β (hpro-IL-1β), but not rhIL-38 protein. Western blotting analysis was performed using rabbit anti-human pro-IL-1β polyclonal Ab (Santa Cruz Biotechnology, Dallas, TX) and rabbit anti-human IL-38 polyclonal Ab (established at our laboratory).

Western blotting analysis showed that human IL-38 cDNA-transfected 293T cells expressed IL-38 protein of approximately 18 kDa in the cells and in the supernatant (lanes 2 and 4 in Fig1A). The molecular mass of recombinant human IL-38 protein fused with a His tag was approximately 20 kDa (Fig. 1A). We established anti-human IL-38 mAbs as reported previously [5], [6]. In this study, we newly established a human IL-38 ELISA system using two anti-human IL-38 mAbs (clone H160A [mouse IgG1], clone H127C [mouse IgG2b]) (Fig. 1B). Sandwich ELISA analysis revealed that the level of IL-38 in the supernatants of human IL-38 cDNA-transfected 293T cells was 39.0 ng/mL. In contrast, IL-38 protein was not detectable in 293T cells transfected with the vector only. Taken together, the data indicated that human IL-38 protein is soluble, despite the fact that the IL-38 gene does not have a signal peptide. It has been reported that, like other IL-1 family cytokines, IL-18 can be cleaved by caspase-1, chymase, and PR3 [18]. Therefore we examined whether caspase-1, chymase, and PR3 can cleave recombinant human IL-38 protein. As shown in Fig. 1C, all three enzymes cleaved recombinant human IL-1β, but not human IL-38.

### 3.2 IL-38 expression in RA serum and synovium

**Fig. 2:**
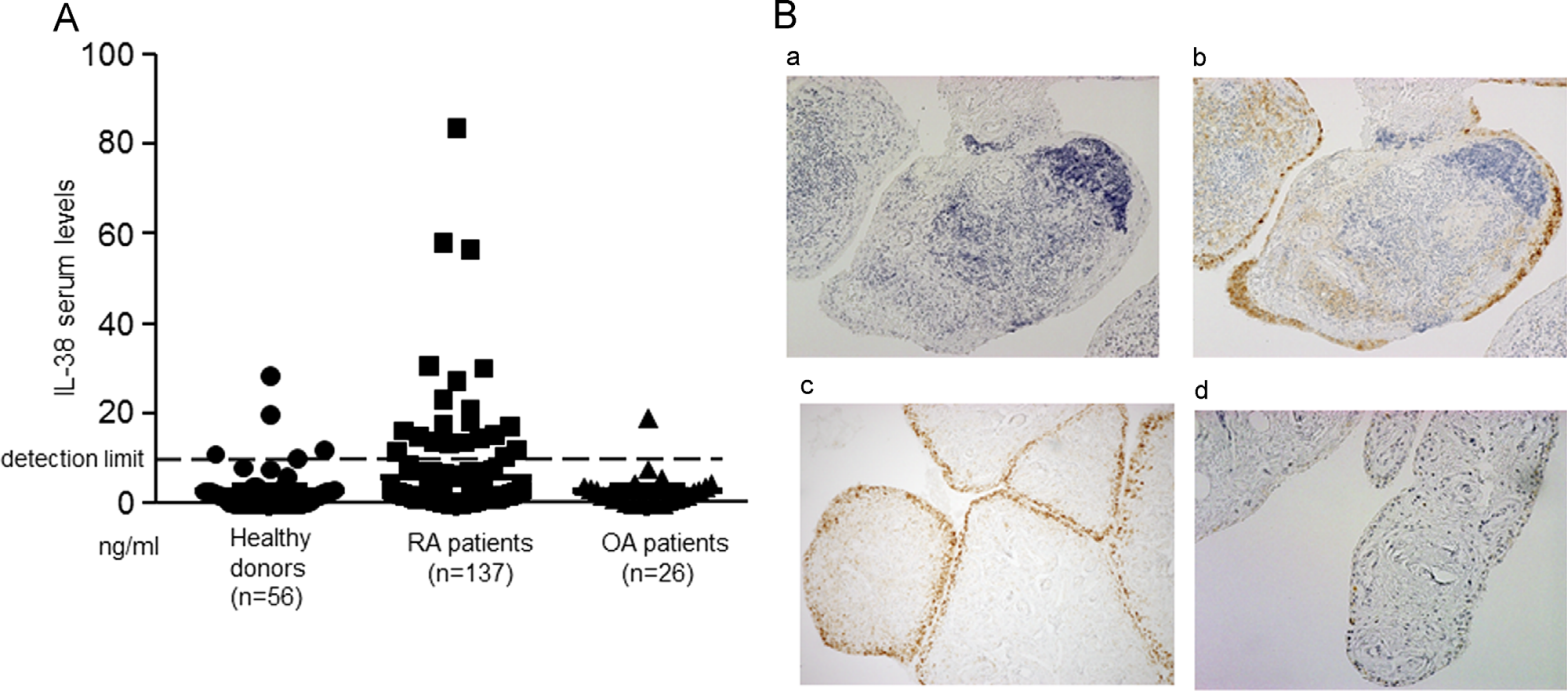
IL-38 expression in RA serum and synovium. (A) Serum levels of IL-38 in RA, OA patients and healthy donors. Serum levels of IL-38 was examined by IL-38 ELISA. Twenty-one of 137 RA (15.3%), one of 26 OA patients (3.9%) and 5 of 56 controls (8.9%) were elevated above the detection limit. Detection limit is 9.35 ng/ml. (B) IL-38 is expressed in RA, but not OA synovium. Synovial tissues were obtained from 7 RA patients and 2 OA patients. Synovial tissues were immunostained with anti-human IL-38 mAb [H127C, mouse IgG2b] (b, c, d) or isotype-matched control mouse IgG2b Ab (a). Representative sections of synovial tissues from 2 RA patients (b, c) and a patient with OA (d) are shown. Original magnification: ×200.

We examined the serum levels of IL-38 in RA patients, OA patients and healthy donors by using ELISA for human IL-38, and the corresponding levels were 5.7±0.94 ng/mL (*n*=137), 2.8±0.876 ng/mL (*n*=26), and 2.8±0.69 ng/mL (*n*=56), respectively. Twenty-one of 137 RA (15.3%) patients, one of 26 OA patients (3.9%), and 5 of 56 controls (8.9%) showed elevated IL-38 levels above the limit of detection (9.35 ng/mL) of our ELISA assay. However, there were no significant differences in serum IL-38 levels among RA patients, OA patients and healthy donors (Fig. 2A). Next, we evaluated IL-38 expression in the synovial tissue of RA and OA patients. Synovial tissue samples from 7 RA patients and 2 OA patients were subjected to immunohistochemical analysis. We found that IL-38 protein was highly expressed in the synovial lining of RA synovium (b, c in Fig. 2B), whereas no immunohistochemical reaction was detected in RA synovium using control mouse IgG2b Ab (a in Fig. 2B). Moreover, IL-38 protein was expressed very weakly in the OA synovium lining (Fig. 2B, d). No immunohistochemical reaction was detected in OA synovium using control Ab (data not shown).

### 3.3 IL-38 gene deficiency exacerbates disease severity in autoantibody-induced arthritis

**Fig. 3:**
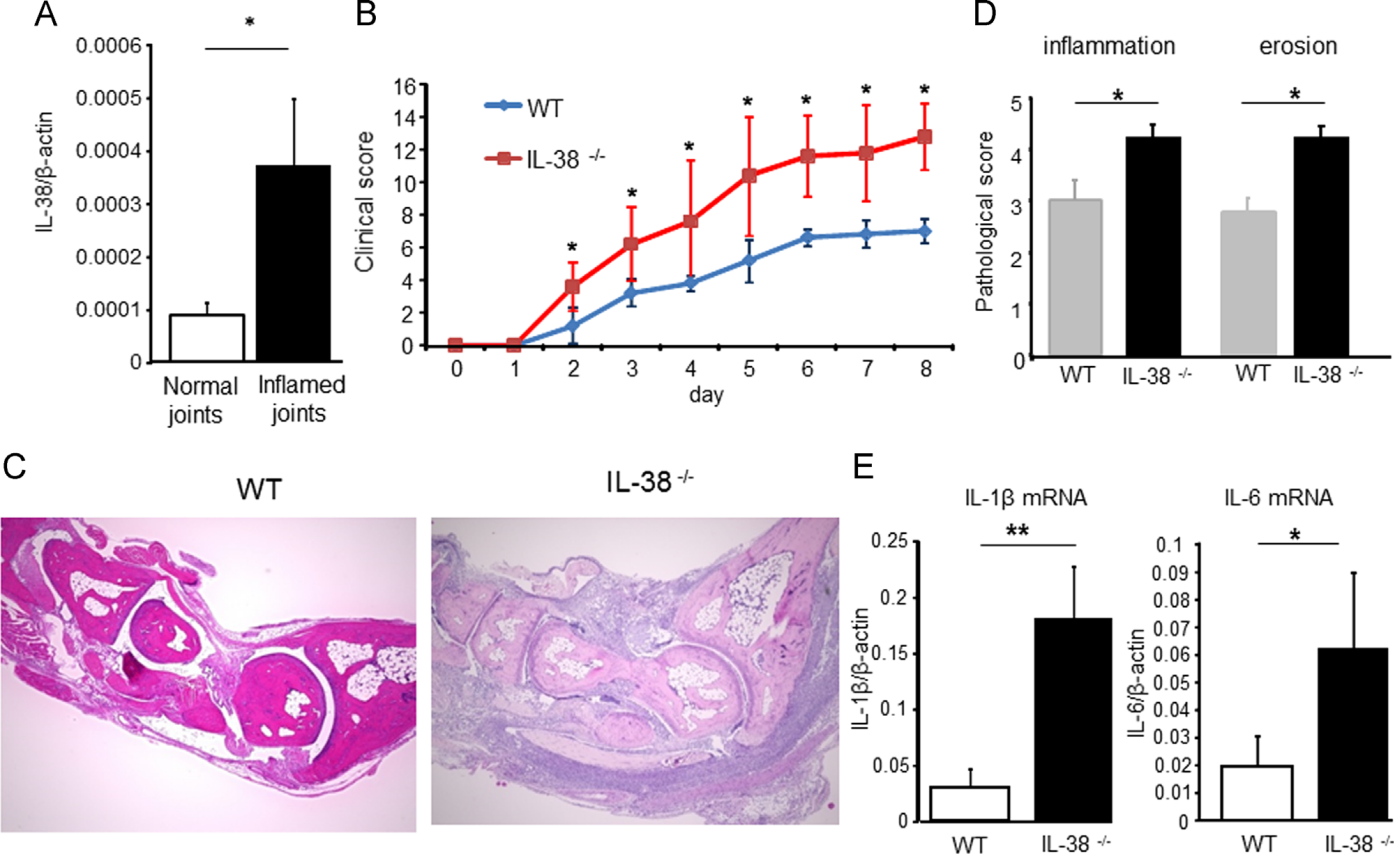
IL-38 gene deficiency enhances joint inflammation in RA mouse model. Arthritis was initiated in female IL-38^−/−^ mice and control B6 WT mice via intraperitoneal administration of K/BxN mouse serum. (A) IL-38 mRNA in ankle joints (8 ankles/group from two separate experiments) was determined before or 8 days after K/BxN serum transfer. *p<0.05, normal vs. inflamed joints. (B) Clinical score of K/BxN serum transfer arthritis in WT and IL-38^−/−^ mice on a 0–16 scale (*n*=5/group), *p<0.05, **p<0.01, WT vs. IL-38^−/−^ mice. Data are representative of at least 2 separate experiments. (C) Histomorphometric quantification of arthritic tissue. Data were pooled in two independent experiments (10 ankles/group from two separate experiments). *p<0.05, WT vs. IL-38^−/−^ mice. (D) Histopathologic findings in the ankle joints of representative WT (left) and IL-38^−/−^ mice (right). Hematoxylin and eosin stained; original magnification ×40. (E) Cytokine mRNA in the ankle joints (10 ankles/group from two separate experiments) was examined at day 7 or 8 arthritis. *p<0.05, WT vs. IL-38^−/−^ mice. Values in A–C and E are the mean±SEM.

The prominent expression of IL-38 protein in synovium of RA patients was confirmed. However, its expression in mouse joints had not been investigated. K/BxN arthritis is an IgG autoantibody-mediated immune complex mouse arthritis that results in marked joint tissue inflammation within a few days after intraperitoneal injection of arthritogenic serum [11]. IL-38 mRNA was significantly increased in mouse joints during autoantibody-induced arthritis (Fig. 3A). To explore the role of IL-38 in a RA mouse model, we induced K/BxN serum transfer arthritis in female B6 mice lacking the IL-38 gene (IL-38^−/−^). IL-38 deficient mice (Il1f10tm1Lex/Mmucd) used in this study, were backcrossed 9 times with C57BL/6NCrl (B6) mice. We did not observe a significant phenotype in C57/BL6 background IL-38 deficient mice until 18 weeks after birth. As controls, we used age- and sex-matched B6 WT mice. We found that IL-38^−^^/−^ mice exhibited significant exacerbation of the clinical scores during arthritis, when compared with control WT B6 mice (Fig. 3B). Histological analysis was performed as shown in Fig. 3C. Histomorphometric quantification of the arthritic changes in the joint tissues confirmed the clinical assessment, with significant increases in inflammation and bone erosion scores in IL-38^−/−^ mice (Fig. 3D). The corresponding expression of mRNA for IL-1β and IL-6 was significantly more up-regulated in the ankle joints obtained from IL-38^−/−^ mice as compared with control mice (Fig. 3E).

## 4 Discussion

IL-38 lacks a signal peptide and has no caspase-1 consensus cleavage site [2]. Our in vitro study showed that human IL-38 protein was not cleaved by recombinant caspase-1, chymase, or PR3 in vitro. However, overexpression of human IL-38 cDNA produced a soluble form of IL-38, as reported previously [1]. We also found that human IL-38 protein was detectable at higher levels in sera of RA patients as well as at lower concentrations in OA patients and normal subjects. Consistent with previous findings [1], our present results show that human IL-38 protein is produced as a soluble form and may function as a secreted ligand. However, we did not examine the natural N terminus of the soluble IL-38, and further analysis will therefore be needed.

Clinical studies using anti-inflammatory cytokine monoclonal antibodies such as anti-TNF-α and anti-IL-6 have revealed that inflammatory cytokines are involved in the development of RA. Moreover, previous studies have shown that recombinant IL-1Ra (anakinra) can be used for treatment of systemic juvenile idiopathic arthritis and a proportion of RA patients [3]. It has been suggested that IL-38 may have an antagonistic role, because of its amino acid homology to naturally occurring IL-1Ra [2]. Recently, a study using an immobilized extracellular IL-36R domain-Fc construct in a low-sensitivity binding assay has suggested that IL-38 binds to the extracellular domain of recombinant IL-1Rrp2 (IL-36R), and this finding will need to be confirmed [3]. In the present study, we showed that synovial tissues obtained from RA patients, but not OA patients, strongly expressed IL-38 protein and that the serum IL-38 levels in RA patients were higher (but not significantly) than those in control subjects. Moreover, disease severity and expression of the IL-1β and IL-6 genes in the IL-38^−/−^ RA model mice were more marked than those in WT mice, suggesting that IL-38 acts as an anti-inflammatory cytokine in our mouse RA model.

Although IL-38 protein has been demonstrated to be expressed in human skin and tonsil [1], we report here that IL-38 protein is also strongly expressed in synovia of RA patients. Synovial tissue-derived IL-38 may play a local role in the development of joint inflammation. In the present study, the soluble form of IL-38 protein was detected in the sera obtained from 15.3% and 3.9% of RA and OA patients, respectively. However, serum levels of IL-38 in RA patients did not correlate with their disease activity, disease duration, or treatment (data not shown). Soluble IL-38 in the serum could be a specific RA diagnostic marker in a subset of patients. In other autoimmune diseases, 7 of 37 SLE (18.9%) and none of 5 adult onset Still's disease patients were elevated above the limit of detection (manuscript in preparation). Further analysis of the role of IL-38 in human autoimmune disease is needed and will require an analysis of larger patient populations.

A study using an antibody-induced arthritis model has shown that IL-1β is required for disease progression [19]. Furthermore, IL-1Ra-deficient mice on a BALB/c background spontaneously develop chronic inflammatory arthropathy [20]. The pathogenic role of the IL-1 pathway has been confirmed in an experimental murine arthritis model. In our model, IL-38 expression was up-regulated, at least at the mRNA level, during arthritis, suggesting that it may participate in the development of arthritis. B6 background IL-38^−^^/−^ mice developed more severe arthritis than control WT mice, associated with enhanced expression of IL-1β and IL-6 in the ankle joints. It has been shown that IL-1Ra and IL-38 inhibit *C. albicans*-induced Th17 production, as well as IL-22 production by PBMC [3]. Like IL-1Ra, IL-38 may have an antagonistic role, in view of its amino acid homology with naturally occurring IL-1Ra [1], [3]. Therefore, IL-38 may attenuate joint inflammation by inhibiting IL-1-induced inflammation. Systemic administration of recombinant mouse IL-38 (1 μg/mouse) did not inhibit arthritis development in the K/BxN arthritis model (data not shown). However, the precise dosage of recombinant IL-38 and the drug administration route required to inhibit arthritis development needs to be confirmed. It is well known that IL-1 family members IL-1β and IL-18 have both inflammatory and anti-inflammatory effects in vivo [18,21]. Especially important, these cytokines have inflammatory and anti-inflammatory effects depending on dose. Whereas IL-38 gene deficiency enhanced arthritis, systemic administration of recombinant IL-38 protein did not inhibit arthritis development. Therefore, it is possible that IL-38 may have dose dependent effects in inflammatory vs. anti-inflammatory responses. Further analysis is required to test this hypothesis.

In summary, IL-38 exerts anti-inflammatory effects in a RA mouse model, and is structurally and functionally related to receptor antagonists, including IL-1Ra.

## Conflict of interest

The authors declare no conflict of interest.
